# A comparison of indices of glucose metabolism in five black populations: data from modeling the epidemiologic transition study (METS)

**DOI:** 10.1186/s12889-015-2233-0

**Published:** 2015-09-15

**Authors:** Yacoba Atiase, Kathryn Farni, Jacob Plange-Rhule, Amy Luke, Pascal Bovet, Terrence G. Forrester, Vicki Lambert, Naomi S. Levitt, Stephanie Kliethermes, Guichan Cao, Ramon A. Durazo-Arvizu, Richard S. Cooper, Lara R. Dugas

**Affiliations:** University of Ghana Medical School, Accra, Ghana; Department of Public Health Sciences, Loyola University Chicago Stritch School of Medicine, Maywood, IL USA; Kwame Nkrumah University of Science and Technology, Kumasi, Ghana; Institute of Social & Preventive Medicine, Lausanne University Hospital, Lausanne, Switzerland; Switzerland & Ministry of Health, Victoria, Republic of Seychelles; Solutions for Developing Countries (SODECO), University of the West Indies, Mona, Kingston, Jamaica; Research Unit for Exercise Science and Sports Medicine, University of Cape Town, Cape Town, South Africa; Department of Medicine, University of Cape Town, Cape Town, South Africa; Public Health Sciences, 2160 S. 1st Ave, Maywood, IL 60153 USA

## Abstract

**Background:**

Globally, Africans and African Americans experience a disproportionate burden of type 2 diabetes, compared to other race and ethnic groups. The aim of the study was to examine the association of plasma glucose with indices of glucose metabolism in young adults of African origin from 5 different countries.

**Methods:**

We identified participants from the Modeling the Epidemiologic Transition Study, an international study of weight change and cardiovascular disease (CVD) risk in five populations of African origin: USA (US), Jamaica, Ghana, South Africa, and Seychelles. For the current study, we included 667 participants (34.8 ± 6.3 years), with measures of plasma glucose, insulin, leptin, and adiponectin, as well as moderate and vigorous physical activity (MVPA, minutes/day [min/day]), daily sedentary time (min/day), anthropometrics, and body composition.

**Results:**

Among the 282 men, body mass index (BMI) ranged from 22.1 to 29.6 kg/m^2^ in men and from 25.8 to 34.8 kg/m^2^ in 385 women. MVPA ranged from 26.2 to 47.1 min/day in men, and from 14.3 to 27.3 min/day in women and correlated with adiposity (BMI, waist size, and % body fat) only among US males after controlling for age. Plasma glucose ranged from 4.6 ± 0.8 mmol/L in the South African men to 5.8 mmol/L US men, while the overall prevalence for diabetes was very low, except in the US men and women (6.7 and 12 %, respectively). Using multivariate linear regression, glucose was associated with BMI, age, sex, smoking hypertension, daily sedentary time but not daily MVPA.

**Conclusion:**

Obesity, metabolic risk, and other potential determinants vary significantly between populations at differing stages of the epidemiologic transition, requiring tailored public health policies to address local population characteristics.

## Background

In line with global predictions, type 2 diabetes, once thought to be uncommon in Africa, is on the increase. In one of the most exhaustive reviews of causes of global mortality, Lozano et. al. found that age related deaths from diabetes alone had nearly doubled during the period between 1990 and 2010. Furthermore, distinct regional patterns of growth for diabetes rates exist, with exceptionally high rates found in the Caribbean and parts of Africa. Of note, urban-dwelling blacks in South Africa experienced a 53 % increase in rates of diabetes between 1990 and 2009 [1, 2].

The rates of increase have also been shown to stratify by socio-economic status (SES), with one study from Africa identifying rates of 4.4, 5.0 and 7.0 % in low, lower-middle, and upper-middle income countries respectively. Such findings are supported by studies with a more localized focus [3]. For example, in Tanzania, less than 1 % of the population was recorded as having diabetes in 1989 [3], however, 11 years later, the prevalence rate had quadrupled to almost 4 % [4]. Similarly, in Nigeria the prevalence rate for diabetes was reportedly 3 % in 1997 [5] and after 16 years had increased to almost 7 % [6]. Two Ghanaian studies from the 1950s and 1960s each documented prevalence rates of less than 1 %; 50 later studies documented show prevalence rates more than 5 % [7]. In Seychelles, the prevalence of diabetes increased by 50 % in 15 years with half of cases attributable to obesity [8, 9], and a marked trend for diabetes to cluster among those of low SES [10]. These trends are in line with global projections that diabetes will increase in prevalence with most of this numerical increase occurring in developing countries, including those on the African continent [11]. This appears to be independent of the Global Human Development Index (HDI) of the country [12].

Some of the traditional risk diabetes factors (e.g. age, lack of physical activity) seem to associate differently in people of African descent. Indeed, some studies suggest that visceral adipose tissue traditionally associated with increased risk for insulin resistance (IR) and diabetes, is lower in black than white women [13, 14] and does not correlate with IR as shown in white women [14]. In the US and South Africa, studies have suggested that other factors typically associated with IR in white populations, including serum triglyceride and HDL-cholesterol levels [15, 16], ectopic fat deposition [15, 17], and adipose tissue inflammation [18] are not significant determinants of IR in black populations. However, IR was associated with triglycerides, HDL, HDL-cholesterol, and CRP among blacks in the Seychelles [19, 20]. These findings suggest that apart from genetic influences [21], environmental and lifestyle factors may also contribute to the increasing prevalence of diabetes in Africa and the diaspora.

As far as we are aware, our study is the first to examine the differences in prevalence of diabetes and its determinants in five cohorts of African descent at differing stages of the epidemiologic transition, as indicated by their HDI rank: rural Ghana, peri-urban South Africa, The Seychelles, urban Jamaica and metropolitan Chicago.

## Methods

Five cohorts of young adult participants in the Modeling the Epidemiologic Transition Study (METS) were analyzed. A full description of the METS measurement and laboratory procedures has previously been published [22]. Briefly, METS is an on-going longitudinal study in five countries: Ghana, South Africa, Seychelles, Jamaica, and the United States (US). Twenty five hundred male and female adults between the ages of 25–45 years were enrolled during January 2010 and December 2011, with 500 per site [22]. Individuals were excluded if they were diagnosed with infectious disease (e.g. HIV-positive), were pregnant or lactating, or were unable to participate in normal physical activities. For the purpose of these analyses, we included only participants with completed measurements of serum insulin, adiponectin and leptin (*n* = 667).

The protocol for METS was approved by the Institutional Review Board of Loyola University Chicago, IL, USA; the Committee on Human Research Publication and Ethics of Kwame Nkrumah University of Science and Technology, Kumasi, Ghana; the Health Sciences Faculty Research Ethics Committee of the University of Cape Town, South Africa; the Board for Ethics and Clinical Research of the University of Lausanne, Switzerland; the National Research Ethics Committee of Seychelles; and the Ethics Committee of the University of the West Indies, Kingston, Jamaica.

### Measurements

All measurements were performed early in the morning at outpatient clinics or testing sites, located within the communities.

### Anthropometrics and body composition

Weight and height were measured as previously described [23] and used to calculate body mass index (BMI, kg/m^2^) and classified participants as normal weight (<25 kg/m^2^), overweight (≥25 and <30 kg/m^2^) and obese (≥30 kg/m^2^), in accordance with international standards.

Body composition was measured using bioelectrical impedance analysis (BIA) and used to estimate fat-free mass (FFM) and fat mass (FM) using an equation previously validated in African-origin populations [24].

### Questionnaires

We obtained a basic health history, with a focus on obesity, cardiovascular conditions, and diabetes. We further assessed individual occupation using an occupation questionnaire from the U.K. National Statistics Socio-economic Classification 2000 edition [25]. We used years of education as a proxy for SES.

### Biochemical measures

Participants were asked to fast from the evening prior to the baseline clinic examination. Fasting blood samples were drawn for analysis of adipose-related hormones and adipocytokines, glucose, and insulin. Fasting plasma glucose (FBG) was measured using the glucose oxidase method at each site at the time of collection. Insulin, leptin and adiponectin from all sites were measured using radioimmunoassay kits at the departmental laboratory at Loyola University Chicago (Linco Research, Inc., St. Charles, MO). Participants were determined to have diabetes using the 2010 American Diabetes Association criteria of a FBG ≥7.0 mmol/L [26].

### Physical activity monitoring

Physical activity (PA) was measured using an accelerometer (Actical, Phillips Respironics, Bend, OR, USA) and has been previously described in detail [22]. Briefly, each participant was asked to wear the accelerometer at all times over 8 days, including during sleep; the only time the monitor should be removed was while bathing, showering, or swimming. For data analysis, raw data downloaded from the accelerometers were first passed through a SAS macro program designed to infer non-wear time from 90 or more minutes of continuous zero activity counts. A valid day of physical activity monitoring was defined as one having 10 or more hours of wear time, i.e. ≥62 % of maximal available wear time. Participant files were included for analysis if they contained four or more valid days, i.e. ≥75 % of maximum number of days. Sedentary, moderate and vigorous activity levels were defined using published cut-points: sedentary <100 counts per minute (cpm), moderate 1535–3959 cpm and vigorous ≥3960 cpm [27, 28]. Data are also presented as 1-min bouts of moderate-to-vigorous activity (MVPA) and sedentary time.

#### Statistical analysis

Standard descriptive statistics were used to summarize the characteristics of participants in each of the five study sites. For continuous measures, we calculated means and standard deviations (e.g. age, weight, % body fat, BMI, minutes of PA, glucose, etc.), and proportions were reported for categorical variables (overweight/obese, female sex, manual labor). The Kernel density plot is used to illustrate the distributions of plasma glucose and BMI by site. Partial Pearson correlation coefficients were used to assess the linear associations between adiposity and measures of physical activity and education as well as between blood glucose levels and measures of adiposity and physical activity. All correlations are adjusted for age and reported by sex and site. A multiple linear regression analysis was used to examine associations between measures of glucose metabolism and independent variables (e.g. age, sex, and site). An alpha of 0.05 was used to denote statistical significance. All statistical analyses were performed using STATA v.12 (College Station, TX, USA).

## Results

The final sample included all participants from the METS pool who had complete laboratory and physical activity measurements and included 667 participants, of whom 282 were men and 385 were women (57.7 %). We analyzed laboratory measurements, measures of adiposity, socioeconomic indicators, and measures of physical activity in two main groups by sex, and five subgroups by site (Ghana, RSA, Jamaica, Seychelles, and US). Men and women were examined separately as a result of their very different body composition data and as previously published [23, 24, 29, 30].

### Participant characteristics

Complete participant characteristics for men and women can be found in Table 1.

**Table 1:** Participant characteristics by site and sex (mean ± SD)

Men	Ghana	South Africa	Jamaica	Seychelles	United States	Total
	(53)	(43)	(59)	(53)	(74)	(282)
Age (y)	36.3 ± 6.6	31.8 ± 6.2*	34.2 ± 5.7	35.2 ± 5.6	35.6 ± 6.6	34.8 ± 6.3
Body Mass Index (kg/m^2^)	22.1 ± 2.5***	22.6 ± 5.9***	23.2 ± 5.0***	25.6 ± 4.3***	29.6 ± 8.0	25.0 ± 6.3
Waist Circumference (cm)	78.5 ± 7.1***	79.1 ± 15.7***	80.0 ± 13.8***	85.5 ± 10.5***	98.5 ± 22.5***	85.5 ± 17.4***
Body Fat (%)	16.0 ± 5.4***	19.5 ± 8.0***	19.5 ± 8.2***	23.0 ± 7.3***	30.9 ± 8.3***	22.5 ± 9.2***
MVPA (min/day, 1-min bouts)	47.1 ± 21.7	53.5 ± 24.5**	26.2 ± 24.5	41.3 ± 27.3	34.8 ± 37.5	39.5 ± 29.7
MVPA (min/day 10-min bouts)	22.2 ± 14.8	28.0 ± 16.3	10.8 ± 19.5	20.9 ± 17.5	21.0 ± 35.1	20.2 ± 23.7
Sedentary (total min for day)	192.0 ± 40.0	204.2 ± 38.6	227.6 ± 61.0**	199.2 ± 44.1	206.0 ± 46.6	206.3 ± 48.4
Education (y)	9.5 ± 3.7***	10.0 ± 2.5***	10.7 ± 2.5***	12.1 ± 2.1	12.8 ± 1.9	11.2 ± 2.9
Manual Laborer (%)	66.7	75.6	62.8	65.9	69.4	67.9
Glucose (mmol/L)	5.6 ± 0.6	4.6 ± 0.8***	5.3 ± 0.5**	5.6 ± 0.8	5.8 ± 1.1	5.5 ± 0.9
Insulin (pmol/L)	79.8 ± 28.1	92.7 ± 109.8	87.0 ± 43.5	96.6 ± 56.7	138.0 ± 91.4	102.0 ± 75.0
Leptin (μg/L)	4.9 ± 6.3**	5.4 ± 8.8*	5.4 ± 8.4**	7.1 ± 6.9	11.2 ± 13.4	7.1 ± 9.6
Adiponectin (μg/mL)	9.2 ± 3.8	9.7 ± 4.3	5.8 ± 4.0	5.3 ± 3.0*	7.7 ± 4.7	7.5 ± 4.4
Log HOMA	1.0 ± 0.4***	0.8 ± 0.6***	1.0 ± 0.6***	1.1 ± 0.6*	1.4 ± 0.7	1.1 ± 0.6
Diabetes (%)	0	0	0	4.0 (*n* = 2)	6.7 (*n* = 5)	2.4 (*n* = 7)
Women	Ghana	South Africa	Jamaica	Seychelles	United States	Total
	(75)	(88)	(70)	(77)	(75)	(385)
Age (y)	35.7 ± 6.2	32.5 ± 6.5*	34.8 ± 5.7	35.7 ± 6.5	35.5 ± 6.0	34.8 ± 6.3
Body Mass Index (kg/m^2^)	25.8 ± 6.2***	31.6 ± 8.5	28.5 ± 6.8***	28.2 ± 5.8***	34.8 ± 9.2	29.9 ± 8.0
Waist Circumference (cm)	85.1 ± 12.7***	97.3 ± 18.4	89.3 ± 13.7***	88.9 ± 11.7***	104.0 ± 20.3	93.1 ± 17.2
Body Fat (%)	35.3 ± 6.3***	42.1 ± 7.2*	38.4 ± 6.5***	38.6 ± 7.0***	45.0 ± 6.2	40.0 ± 7.5
MVPA (min/day, 1-min bouts)	27.3 ± 18.4***	19.9 ± 15.6	14.6 ± 10.9	20.3 ± 11.8	14.3 ± 16.3	19.4 ± 15.6
MVPA (min/day, 10-min bouts)	12.2 ± 11.5***	9.2 ± 10.5	5.5 ± 6.7	8.2 ± 8.6	5.9 ± 10.3	8.3 ± 9.9
Sedentary time (total min for day)	189.4 ± 33.6	221.1 ± 44.5	207.5 ± 47.5	191.5 ± 46.2	212.6 ± 43.3	205.2 ± 45.7
Education (y)	7.0 ± 4.2***	10.0 ± 2.3***	11.3 ± 2.3***	13.1 ± 2.4	13.4 ± 2.6	10.9 ± 3.7
Manual Laborer (%)^a^	93.0	87.0	61.0	15.2	33.3	58.7
Glucose (mmol/L)	5.5 ± 0.6	4.6 ± 0.8***	5.1 ± 0.5	5.4 ± 1.4	5.5 ± 1.9	5.2 ± 1.1
Insulin (pmol/L)	103.3 ± 45.8***	157.1 ± 96.3	131.1 ± 62.4**	133.1 ± 77.3**	175.6 ± 86.0	140.7 ± 80.0
Leptin (μg/L)	26.5 ± 21.0***	26.5 ± 21.0	35.5 ± 22.1	28.3 ± 20.6**	41.4 ± 19.2	32.8 ± 21.6
Adiponectin (μg/mL)	10.2 ± 4.3	12.0 ± 6.5*	8.2 ± 5.3	7.2 ± 4.5	9.2 ± 5.8	9.6 ± 5.6
Log HOMA	1.2 ± 0.5***	1.3 ± 0.7***	1.3 ± 0.5**	1.3 ± 0.6**	1.7 ± 0.5	1.4 ± 0.6
Diabetes (0 = no;1 = yes)	1.0 (*n* = 1)	2.2 (*n* = 2)	1.4 (*n* = 1)	2.6 (*n* = 2)	12.0 (*n* = 9)	3.9 (*n* = 15)

Significantly different from USA * *p* < 0.05, **p < 0.01, ***p < 0.001, ^a^ significantly different across sites *p* < 0.001, chi-squared test

Men: The mean age ranged from 31.8 ± 6.2 y in South Africa to 36.3 ± 6.6 y in Ghana. Measures of adiposity followed a predictable pattern overall, with mean BMI, waist circumference, fat mass, and body fat percentage at their highest in US and lowest in Ghanaian subjects. Among the Ghanaians, only 2 % were classified as obese, while among the US, an astonishing 42 % were obese. The average years of education ranged from 9.5 ± 3.7 y in Ghana to 12.8 ± 1.9 y in the US. The percentage of participants engaged in manual occupations ranged from 62.8 % in Jamaica to 75.6 % in South Africa, where the primary occupation was construction work.

Women: The mean age ranged from 32.5 ± 6.5 y in South Africa to 35.7 ± 6.5 y in the Seychelles. Not surprisingly, women had significantly higher measures of adiposity compared to their male counterparts. Similarly, measures of adiposity followed a predictable pattern, with the mean BMI, waist circumference, fat mass, and body fat percentage highest among the US participants and lowest among the Ghanaians. Approximately 17 % of Ghanaian women were classified as obese compared to 68 % in the US. The average years of education ranged from 7.0 ± 4.2 y in Ghana to 13.4 ± 2.6 y in the US. The prevalence of manual occupations varied much more widely among the women compared to the men, with approximately 15.2 % of women in the Seychelles performing manual labor, compared to 93 % in Ghana.

### Laboratory measures

Table 1 presents the laboratory measures. Among the men, mean FBG levels were lowest in South Africa (4.6 ± 0.8 mmol/l) and highest within the US (5.8 ± 1.1 mmol/l). The log of HOMA-IR, a measure of insulin resistance, followed a similar pattern to glucose, with the lowest levels among men from South Africa (0.76 ± 0.62) and highest among the US men (1.4 ± 0.7). The Ghanaian men had the lowest mean insulin and leptin levels (79.8 ± 28.1 pmol/L and 4.9 ± 6.3 μg/L, respectively), while the US men had the highest insulin, leptin, as well as percentage participants with diabetes (138.0 ± 91.4 pmol/L, 11.2 ± 13.4 μg/L, and 6.7 %, respectively). Adiponectin levels ranged from 5.3 ± 3.0 μg/mL in Seychelles men to 9.7 ± 4.3 μg/mL in South Africans. Overall the prevalence of diabetes was low among our participants (3.5 %) and did not differ between men and women. However US men and women had significantly more diabetic participants compared to other sites (*p* < 0.05 and *p* < 0.01, respectively).

Within the female participants, these patterns remained consistent. Mean FBG levels were lowest in South Africa 83.2 ± 15.0 mg/dL to 98.9 ± 26.6 mg/dL in the US. Unlike the men, the women’s measures of HOMA-IR were consistent with glucose patterns across sites: HOMA-IR was lowest within the Ghanaian women (1.16 ± 0.51) and highest in the US women (1.65 ± 0.52). Leptin, insulin, and percentage of diabetes were similarly lowest in Ghanaians (26.5 ± 21.0 μg/L, 100.1 ± 44.3 pmol/L, and 1 %, respectively) and highest in the US (41.4 ± 19.2 μg/L, 170.0 ± 83.2 pmol/L, and 13 %, respectively). Mean adiponectin levels were lowest in Seychelles (7.2 ± 4.5 μg/L) and highest in South Africa (12.0 ± 6.5 μg/L).

**Fig. 1:**
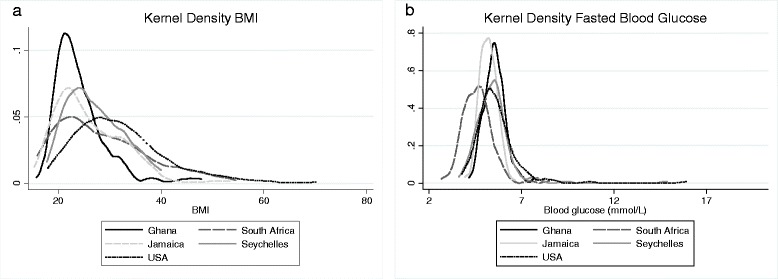
Kernel density plot indicating BMI by site for men (1**a**) and women (1**b**)

Figure 1 presents the Kernel Density plots for BMI (kg/m^2^) and FBG (mmol/L), by site. For BMI (1a), it can be seen that the density is most different between Ghana and the US, whereby the distribution for participants from Ghana is tighter than the wider spread observed among the US participants. Further, the curve is shifted towards the right for the US, indicating a higher mean BMI. For FBG, it can be similarly observed that the density for the data from participants from Ghana is tighter than the wider distribution for the US participants.

### Physical activity

Men: The mean number of MVPA in 1-min bouts ranged from 26.2 ± 24.5 min/day in Jamaica to 53.5 ± 24.5 min/day in South Africa, and 10-min bouts ranged from 10.8 ± 19.5 min/day in Jamaica to 28.0 ± 16.3 min/day in South Africa. The total sedentary time ranged from 192.0 ± 40.0 min/day in Ghana to 227.6 ± 61.0 min/day in Jamaica. Overall, the South African men were the most physically active by our measures, while those from Jamaica were the least physically active and had the largest amount of sedentary time compared to all other sites.

Women: The number of MVPA minutes in 1-min bouts ranged from 14.3 ± 16.3 min/day in the US to 27.3 ± 18.4 min/day in Ghana, and 10-min bouts ranged from 5.5 ± 6.7 min/day in Jamaica to 12.2 ± 11.5 min/day in Ghana. The total sedentary time ranged from 189.4 ± 33.6 min/day in Ghana to 221.1 ± 44.5 min/day in South Africa. Unlike their male counterparts, the Ghanaian women were the most physically active overall, while those from the US and Jamaica appeared to be the least physically active and most sedentary.

### Correlations

Partial Pearson correlations were examined separately in men and women between measures of adiposity (BMI, waist circumference, and percent body fat) physical activity and SES (Table 2). All correlations were adjusted for age to account for any age-related physical activity effects. Within the men from Ghana, South Africa, and Jamaica, we could account for no statistically significant correlations between any of the measures of physical activity or sedentary time with measures of adiposity (*p* > 0.05 for all). Among the Seychellois, adiposity measures were significantly, albeit weakly, negatively correlated with physical activity in both 1 and 10-min bouts, with the exception of BMI and 10-min bouts of physical activity which was not significant. Likewise, among the US participants, adiposity was significantly negatively correlated with measures of physical activity (*p* < 0.05 for all). Sedentary time did not correlate with adiposity within any of the sites. Among women, the significant correlations between physical activity, sedentary time, and adiposity were considerably fewer.

**Table 2:** Partial correlation coefficients controlled for age between adiposity and measures of physical activity and education by site and sex

	Ghana			South Africa			Jamaica			Seychelles			United States		
	(53)			(43)			(55)			(50)			(74)		
Men	BMI	Waist	% Fat	BMI	Waist	% Fat	BMI	Waist	% Fat	BMI	Waist	% Fat	BMI	Waist	% Fat
MVPA (min/day in 1-min bouts)	−0.15	−0.11	−0.01	−0.17	−0.01	−0.06	−0.02	0	−0.05	−0.31*	−0.31*	−0.36*	−0.25*	−0.24*	−0.28*
MVPA (min/day in 10-min bouts)	−0.06	−0.18	−0.20	−0.05	−0.14	−0.23	0	−0.1	−0.09	−0.28	−0.36**	−0.41**	−0.23*	−0.24*	−0.27*
Sedentary Time (min/day in 1-min bouts)	−0.07	−0.09	0.05	−0.06	−0.02	0.07	−0.07	−0.06	−0.08	−0.05	−0.10	−0.16	−0.1	−0.10	−0.11
Education (y)	0.07	0.13	0.09	0.1	0.07	0.05	0.15	0.17	0.20	0.15	0.24	0.18	0.11	0.05	0.11
	Ghana			South Africa			Jamaica			Seychelles			United States		
	(74)			(87)			(62)			(71)			(72)		
WOMEN	BMI	Waist	% Fat	BMI	Waist	% Fat	BMI	Waist	% Fat	BMI	Waist	% Fat	BMI	Waist	% Fat
MVPA (min/day in 1-min bouts)	0.06	−0.03	−0.08	−0.21*	−0.16	−0.13	−0.11	−0.03	−0.13	0.06	−0.02	−0.03	−0.23*	−0.18	−0.26
MVPA (min/day in 10-min bouts)	−0.07	0.08	0.01	−0.19	−0.15	−0.12	−0.06	−0.10	−0.22	−0.05	0	−0.06	−0.2	−0.22	−0.25*
Sedentary Time (min/day in 1-min bouts)	0.09	−0.07	0.09	−0.13	−0.26*	−0.23	−0.21	−0.23	−0.21	−0.2	−0.26*	−0.18	0.1	−0.06	−0.06
Education (y)	−0.2	−0.24*	−0.22	−0.05	−0.13	0	0	−0.02	0.10	−0.12	−0.15	−0.01	−0.06	−0.08	−0.06

* *p* < 0.05, ** *p* < 0.005, *** *p* < 0.001

Years of education was not correlated with any measures of adiposity except for waist size among Ghanaian women, who showed a small but significant negative correlation (*p* < 0.05) (Table 3). Likewise, the only significant correlation between years of education and blood glucose levels after adjusting for age were among Jamaican women (0.24, *p* < 0.05 and US men (−0.27, *p* < 0.05) (Table 3).

**Table 3:** Partial correlation coefficients controlled for age between blood glucose levels and measures of adiposity, physical activity, education, and adipokines by site and sex

	Ghana		South Africa		Jamaica		Seychelles		United States	
	Men	Women	Men	Women	Men	Women	Men	Women	Men	Women
Sample Size	53	75	43	88	58	70	53	77	74	75
BMI	0.13	−0.02	0.15	0.23*	−0.01	0	0.17	0.13	0.31*	−0.06
Waist Circumference	0.16	0.07	0.14	0.29*	−0.05	−0.02	0.31*	0.18	0.33**	0.01
Percent Body Fat	0.19	−0.04	0.21	−0.17	−0.13	−0.10	0.24	0.14	0.33**	−0.01
Education (y)	−0.16	−0.05	−0.08	−0.14	0.13	0.24*	0.16	−0.14	−0.27*	−0.11
Sample size	49	70	43	88	54	62	50	71	70	72
MVPA (min/day in 1-min bouts)	0.23	0.01	−0.03	−0.19	−0.05	−0.25*	−0.35*	−0.05	0.15	−0.09
MVPA (min/day in 10-min bouts)	0.18	0.02	−0.09	−0.11	−0.10	−0.25*	−0.27	0.03	0.16	0
Sedentary Time (min/day in 1-min bouts)	−0.33*	−0.31*	0	−0.07	−0.25	−0.08	−0.07	−0.33*	−0.32*	−0.18
Sample size	53	75	42	88	55	65	46	63	67	61
Leptin (μg/L)	0.04	0.01	0.14	0.07	−0.04	−0.08	0.07	0	0.14	−0.15
Adiponectin (ng/mL)	0.07	−0.02	0	−0.30**	0.07	−0.21	−0.19	0.01	−0.30*	−0.04
Insulin (pmol/L)	0.04	0.21	0.12	0.32**	0.19	0.06	0.28*	0.33**	0.35**	0.01

* *p* < 0.05, ** *p* < 0.005, *** *p* < 0.001

Correlations between blood glucose levels and adiposity, physical activity, and hormone profiles were also examined among men and women across sites (Table 3). Among men, blood glucose levels were positively associated with all measures of adiposity (BMI, waist circumference, and percent body fat) when controlling for age in the participants from the US. The only other site that showed a relationship between adiposity and glucose was the sample of men from Seychelles, with a significant correlation between waist circumference and glucose of 0.31 (*p* < 0.05), after adjusting for age. Likewise, women showed few statistically significant correlations between glucose and measures of adiposity, with the participants from South Africa being the only exception.

As with correlations between physical activity and adiposity, significant correlations between physical activity and blood glucose levels, adjusting for age, were limited to a few subgroups. For example, Seychellois men and Jamaican women both showed statistically significant negative correlations between physical activity and glucose (*p* < 0.05), but no significant results were seen in any other subgroups. Interestingly, sedentary time was significantly negatively associated with glucose levels in men and women in Ghana, women in Seychelles, and men in the US (*p* < 0.05).

Leptin was not statistically significantly associated with glucose levels in any of the subgroups. Adiponectin levels were significantly and negatively associated with glucose levels within two subgroups: South African women and US men (−0.30, *p* < 0.05).

### Regressions

We performed a multiple linear regression analysis to examine the association of BMI, with blood glucose, adjusting for age, sex, and years of education, smoking, hypertension, daily MVPA and sedentary time (Table 4). Participants with type 2 diabetes were excluded from this analysis (*N* = 22). BMI was positively associated with blood glucose levels and only participants from South Africa had mean blood glucose levels lower than the US participants (0.64 mmol/L, *p* < 0.001). Education, was negatively associated with glucose levels, yet these results trended non-significantly (*p* = 0.213). Surprisingly, only sedentary time and not MVPA was associated with lower FBG.

**Table 4:** The association between fasting glucose level and determining factors according to multivariate regression. Participants with type 2 diabetes (*N* = 22) were excluded

	Beta (S.E.)	95 % CI	*p*-value
BMI (kg/m2)	0.01 ± 0.01	(0.01–0.02)	<0.001
Age (y)	0.02 ± 0.004	(0.01–0.03)	<0.001
Sex (male = 0; female = 1)	−0.22 ± 0.09	(−0.39–0.16)	<0.001
Smoker (no = 0; yes = 1)	−0.09 ± 0.02	(−0.14–0.05)	<0.001
Hypertension (no = 0; yes = 1)	0.18 ± 0.10	(0.05–0.30)	0.005
Education (years)	−0.01 ± 0.001	(−0.03–0.01)	0.213
MVPA (min/day)	−0.002 ± 0.001	(−0.004–0.001)	0.190
Sedentary time (1-min bouts)	−0.002 ± 0.001	(−0.004–0.001)	0.001
Site			
Ghana	0.36 ± 0.10	(0.16–0.56)	0.001
South Africa	−0.64 ± 0.08	(−0.80–0.47)	<0.001
Jamaica	−0.01 ± 0.08	(−0.15–0.17)	0.898
Seychelles	0.17 ± 0.08	(0.01–0.32)	0.037

## Discussion

To summarize, this study examined the relationship between obesity, physical activity, SES, adipokines and fasted blood glucose levels in five heterogeneous cultural settings, adding to a growing body of literature exploring the effect of country-level development and the development of chronic disease [2–4, 8, 10, 14, 31–35]. We found that FBG levels were correlated with adiposity in US men almost exclusively, with additional correlations present between individual levels of adiposity and glucose in South African women (BMI and waist circumference) and Seychellois men (waist circumference). Glucose was negatively correlated with adiponectin levels among South African women and US men only. Glucose levels had a weak inverse correlation with measures of physical activity among Jamaican women and Seychellois men, but physical activity levels across all groups were strikingly low. In a regression analysis, glucose was strongly linearly associated with BMI, age, and sex after adjusting for confounding variables. Education, was negatively associated with glucose levels, yet these results trended non-significantly. Overall, the relationships between glucose and measures of adiposity, physical activity, and SES demonstrated remarkably site and sex specific profiles. Perhaps not surprisingly, the subsample of men from the US tended to fit most closely with popular conceptions of the relationships between adiposity, glucose, education, and physical activity levels, i.e. higher levels of adiposity, lower levels of physical activity, and lower SES are associated with higher glucose levels [36–38]. The variation in these relationships within other subgroups indicates that these concepts almost certainly require interpretation through a site-specific lens. Indeed Shrivastava et al. [39] reported disparate diabetes prevalence in divergent socio-economic communities in India, supporting a community-specific approach.

As expected, measures of adiposity tracked closely with each site’s HDI rank, with the exception of South African women, whose levels of overweight and obesity were higher than those of Jamaican and Seychellois women. Interestingly, although socioeconomic status correlates well with years of education [33], and patterns of adiposity correlated with HDI status between groups, we did not find a significant and robust correlation between education and measures of adiposity in any subgroups. However, this may reflect lack of power as both job category and education were strongly related to obesity (in opposite directions in men and women) in a larger population based sample in the Seychelles [40]. Therefore, socioeconomic status was a predictor of between groups but not within group differences in adiposity.

Likewise, measures of physical activity were generally highest among men from lower HDI countries like Ghana and South Africa, and were lower among women and men from higher HDI countries. Notable exceptions to the pattern were Jamaican men, who had low levels of physical activity and reciprocally high levels of sedentary time, yet were relatively lean. However, perhaps the most surprising finding regarding physical activity was its lack of correlation with measures of adiposity or glucose levels within almost every subgroup except for US men. This contrasts with the findings of Luke et al. [23], who found that METS participants with greater adiposity tended to engage in significantly less physical activity. These findings were further supported by the lack of a statistically significant linear relationship between physical activity and glucose levels when levels were examined in a multivariate linear regression controlling for BMI, age, sex, and years of education.

Adipokine associations with measures of glucose were also widely variable. Leptin showed no significant correlation with blood glucose levels in either sex at any site. Leptin levels are widely known to correlate with levels of adiposity, and so it is not necessarily surprising that given the absence of a correlation between adiposity and blood glucose levels in our study, leptin did not demonstrate any association with blood glucose levels. Adiponectin, although strongly inversely correlated with many associated conditions of the metabolic syndrome, showed a negative correlation with glucose levels only among women from South Africa and men from the US.

The sex differences in the relationships between adiposity and SES are most striking in those countries further along in their economic transition, such as Jamaica and the Seychelles. Rossi et al. found that in Seychellois men, SES was directly related to BMI. However, in women, the opposite was true, with increasing SES associated with a decreased BMI. Additionally, the presence of an inverse relationship between SES and BMI within women tended to be a leading indicator of a population-wide metabolic transition [34, 41]. These sex differences have also been reported in Jamaica, where income is inversely related to rates of obesity in women, yet is directly related to rates of obesity in men [42]. In this study and others, Jamaican women tend to be more educated, more obese, and have lower levels of physical activity when compared to men [42]. Our study found the same pattern for Seychellois and US women, but a slightly different profile in the women from Ghana and South Africa, who tended to be less educated and more likely to be employed in manual labor than their male counterparts, yet had higher rates of obesity and lower levels of physical activity.

The effects of obesity, physical activity and SES on adipokine profiles are less well understood, and data is sparse on site-specific profiles from areas in this study. Meilleur et al. found a negative association between adiponectin, adiposity, and insulin resistance when controlling for age and sex within Nigerian and Ghanaian individuals [43]. In Jamaicans, Boyne et al. found that adiponectin levels increased with age and female sex, and decreased with increasing levels of abdominal fat. In Seychelles, the association between CRP and BMI almost disappeared when controlling for leptin [44]. Yet despite women being more obese and insulin resistant, the study found that women had higher adiponectin levels than their male counterparts, likely due to the relatively higher amounts of subcutaneous fat in women [45]. Our study found significant negative correlations between adiponectin and measures of adiposity in every subpopulation except US women and Seychellois men when controlling for age. However, we found no statistically significant correlations between adiponectin and blood glucose when controlling for age except in South African women and US men.

Our study is not without limitations; it should be noted that the data presented here are cross-sectional, and as such no inferences about the direction of associations can be determined. Furthermore, the sample size is limited and caution should be taken when interpreting the results from our study. Finally, while the samples are representative of the communities in which the participants live, they may not be representative of the countries *per se*, and therefore caution should be taken with over interpretation of the data found in our study.

## Conclusions

In conclusion, despite the modest number for significant correlations between these measures within sites, when taken together as a larger cross-cultural study, the data illustrate five unique populations whose health and economic characteristics shape dramatically different between group differences. As such, obesity, metabolic risk, and other potential determinants vary significantly between populations at differing stages of the epidemiologic transition, requiring tailored public health policies to address local population characteristics. Finally, the METS protocol involves a longitudinal component, and so future follow-up data should be probed to discern any changes that occur over time.

## Abbreviations

METS: Modeling the Epidemiologic Transition Study
US: United States
RSA: South Africa
y: Year
BMI: Body mass index
MVPA: Moderate & vigorous physical activity
Min/day: Minutes/day
SES: Socio-economic status
HDI: Human development index
IR: Insulin resistance
HDL: High density lipoprotein
CRP: C-Reactive protein
HIV: Human immunodeficiency virus
FFM: Fat free mass
FM: Fat mass
FBG: Fasted plasma glucose
Cpm: Counts per minute
